# Prognostic value of residual cancer burden after neoadjuvant chemotherapy in breast cancer: a comprehensive subtype-specific analysis

**DOI:** 10.1038/s41598-025-98176-9

**Published:** 2025-04-22

**Authors:** Soo-Young Lee, Tae-Kyung Yoo, Sae Byul Lee, Jisun Kim, Il Yong Chung, Beom Seok Ko, Hee Jeong Kim, Jong Won Lee, Byung Ho Son

**Affiliations:** 1 Department of General Surgery, Inha University College of Medicine, Inha University Hospital, Incheon, Korea; 2 Division of Breast Surgery, Department of Surgery, University of Ulsan College of Medicine, Asan Medical Center, 88, Olympic-Ro 43-gil, Songpa-Gu, Seoul, 05505 Korea

**Keywords:** Neoadjuvant chemotherapy, Residual cancer burden, Subtypes, Overall survival, Disease-free survival, Breast cancer, Outcomes research

## Abstract

This study evaluated the prognostic impact of residual cancer burden (RCB) on breast cancer subtypes following neoadjuvant chemotherapy (NAC). We retrospectively examined 2,416 breast cancer patients treated with NAC and surgery at Asan Medical Center (2015–2020). Baseline characteristics, clinicopathological parameters, recurrence, and survival outcomes were analyzed using Kaplan-Meier and Cox regression methods to assess RCB’s prognostic significance across subtypes. Pathologic complete response (pCR) was achieved in 25.6% (619) of patients. RCB2 was the most common (44.0%, 1,063), followed by RCB3 (19.6%, 474) and RCB1 (10.8%, 260). Among HR-/HER2 + patients, 67% had RCB0/1, while 87% of HR+/HER2- patients had RCB2/3. Higher RCB was significantly associated with worse overall survival (OS) and disease-free survival (DFS) across all subtypes. Subtype-specific analysis revealed that HR-/HER2 + patients with RCB3 and HR-/HER2- patients with RCB2/3 had significantly worse OS and DFS. Multivariate analysis revealed that RCB2/3 (vs. RCB0), total mastectomy (vs. breast-conserving surgery), axillary lymph node dissection (ALND), lymphovascular invasion (LVI), high Ki-67 index (≥ 20), HR negativity, and HER2 negativity were linked to higher risks of recurrence and death (*p* < 0.05). Factors associated with higher RCB included ALND, LVI, higher Ki-67, and HR+/HER2- subtype. RCB classification is a strong prognostic indicator across all subtypes. Patients with RCB2/3 in the HR-/HER2- and RCB3 in the HR-/HER2 + subtypes had particularly poor outcomes, suggesting benefits from additional treatments beyond standard care.

**Supplementary Information:**

The online version contains supplementary material available at 10.1038/s41598-025-98176-9.

## Introduction

Neoadjuvant chemotherapy (NAC) has become a standard treatment for many patients with early-stage breast cancer. It improves outcomes, facilitates breast-conserving surgery (BCS), and reduces the need for extensive axillary management by downstaging the tumor^1^. NAC also allows for monitoring treatment response both radiologically and pathologically, enabling adjustments to subsequent systemic therapy based on response.

Pathologic complete response (pCR), defined as the absence of invasive tumor in the breast and axillary lymph nodes (ypT0 + ypN0 and ypT0/is + ypN0), indicates a favorable response to NAC. Achieving pCR is associated with improved recurrence-free and overall survival (OS), particularly in aggressive tumor subtypes^2–4^. pCR is widely used as an endpoint in neoadjuvant therapy clinical trials. Given the use of pCR as a surrogate regulatory endpoint, both the US Food and Drug Administration and the European Medicines Agency announced between 2012 and 2014 that they would consider accelerated approval for new drugs used in NAC for breast cancer^5^. Consequently, the presence of pCR is guiding decision for adjuvant therapy after NAC. However, pCR offers prognostic information in a binary manner and occurs in only 20–30% of patients. For the remaining 70–80% who have residual disease after NAC, a quantitative assessment of residual cancer in the breast and axillary lymph nodes is required.

The residual cancer burden (RCB) method developed in 2007 comprised measuring the size of the initial tumor bed, cellularity of the invasive lesion of the residual tumor, size of the largest metastasis, and number of metastatic lymph nodes^6^. Two empirically derived cutoff points (1.36 and 3.28) were applied to the continuous RCB score to define four classes: RCB0 (pCR), RCB1 [minimal residual disease (RD), RCB score ≤ 1.36], RCB2 (moderate RD, 1.36 < RCBscore ≤ 3.28), and RCB3 (extensive RD, RCB score > 3.28)^6^. This classification creates the possibility of assessing recurrence risk and guiding individualized treatment options according to the extent of RD. The prognostic value of RCB has been studied using single-center, multicenter, and pooled analyses^7^. In a pooled analysis of 12 independent patient cohorts, RCB demonstrated prognostic value irrespective of pretreatment clinical and pathological features, including hormone receptor (HR) and human epidermal growth factor receptor 2 (HER2) status. The possibility of recurrence correlates with the extent of RD, regardless of the breast cancer subtype^7^. The American Joint Committee on Cancer (AJCC) (8th ) recommended using the RCB method as complementary to post-neoadjuvant pathologic staging and National Comprehensive Cancer Network presented that pathologic evaluation of surgical specimens after NAC should follow the RCB methods as category 2B evidence^8,9^. However, it is neither mandatory nor encompassed in the decision-making process of current guidelines, partly due to lack of guideline enforcement and the need for further validation studies^10,11^. Therefore, further studies, including ours are necessary to provide clinical evidence supporting the prognostic and clinical utility of RCB.

Given the variability in pCR rates among the different subtypes, the prognostic value of RCB should be evaluated in a subtype-specific context. Patients with RCB1 and HR+/HER2- subtypes and patients with pCR have similar EFS. In contrast, patients with the RCB1 and HR-/HER2- subtypes have different prognoses compared to patients with pCR^7^. Breast cancer subtypes affect prognosis differently, depending on the extent of residual cancer. Triple-negative breast cancer (TNBC) is known for its aggressive behavior, increased metastatic potential, and overall poorer prognosis compared to other breast cancer subtypes^12^. In the KEYNOTE-522 (NCT03036488) trial, adding pembrolizumab to chemotherapy in patients with early TNBC resulted in a survival benefit, and patients with RCB3 had poor prognosis irrespective of pembrolizumab use^13^. Clinical data from a large center offers valuable insights for accurately estimating individual recurrence risk, considering both breast cancer subtype and RCB. By conducting a thorough subtype-specific analysis of RCB, we aimed to identify patients who would most benefit from post-neoadjuvant treatment, thereby refining treatment options based on real-world evidence.

## Methods

### Patient and data collection

This retrospective study included breast cancer patients who underwent NAC followed by standard surgery—either BCS or total mastectomy (TM) with axillary staging surgery—at Asan Medical Center between January 2015 and November 2020. Baseline patient characteristics, including age at surgery, body mass index, breast surgery type and clinical stages were collected from the medical records. Pathologic data, including histological and nuclear grade, lymphovascular invasion (LVI), Ki-67 proliferation index, HR and HER2 status and RCB were obtained from the pathologic reports. The formula for calculating RCB is provided in Supplementary Fig. 1. We assessed estrogen and progesterone receptor status using the semiquantitative Allred scoring system. This system combines the percentage of immunohistochemically stained nuclei (0, < 1%, 1–10%, 11–33%, 34–66%, and > 67%) with the average intensity of immunoreactivity (scored as 0, 1, 2, or 3) to produce a final score ranging from 0 to 8. Scores 0–2 were defined as negative, while scores ≤ 3 were considered positive. We categorized the molecular subtypes into four groups: HR+/HER2−, HR+/HER2+, HR−/HER+, and HR−/HER2 − groups.

NAC regimens varied by breast cancer subtype. Patients with HR+/HER2- and HR-/HER2- subtypes primarily received anthracycline-taxane-based regimens, while those with HER2-positive subtypes predominantly received anti-HER2 therapy in combination with chemotherapy. Detailed regimen distributions are provided in Supplementary Table 2. Follow-up data regarding adjuvant chemotherapy, radiotherapy, endocrine therapy (ET), and the dates of recurrence and death were reviewed. The Institutional Review Board of Asan Medical Center approved this study (2017 − 1341).

### Statistical analysis

In Table 1, continuous variables were expressed as means ± standard deviation (SD). To compare differences among RCB classes, we used t-tests for normally distributed continuous variables and Wilcoxon rank-sum tests for non-normally distributed continuous variables. Categorical variables were analyzed using the chi-square test or Fisher’s exact test, as appropriate. The normality of continuous variables was assessed using the Shapiro-Wilk test.

**Table 1:** Baseline patient characteristics and treatment factors according to RCB classes.

	RCB1	RCB2	RCB3	*p*-value
Number of patients	260	1063	474	N/A
Patient characteristics				
Age at initial surgery (years), mean ± SD	48.3 ± 9.7	47.2 ± 9.8	48.9 ± 10.0	0.006
Body mass index (kg/m^2^), mean ± SD	23.5 ± 3.0	23.9 ± 3.8	24.6 ± 4.3	0.003
Clinical T stage				< 0.001
Tis	0	2(0.2)	0	
T1	24(9.2)	87(8.2)	27(5.7)	
T2	175(67.3)	693(65.2)	256(54.0)	
T3	50(19.2)	227(21.4)	133(28.1)	
T4	11(4.2)	54(5.1)	58(12.2)	
Clinical N stage				< 0.001
N0	0	2(0.2)	0	
N1	117(45.0)	479(45.1)	246(51.9)	
N2	19(7.3)	81(7.3)	40(8.4)	
N3	43(16.5)	174(16.4)	135(28.5)	
Subtype				< 0.001
HR+/HER2-	66(25.5)	544(51.2)	313(66.0)	
HR+/HER2+	92(65.5)	173(16.3)	43(9.1)	
HR-/HER2+	46(17.8)	101(9.5)	32(6.8)	
HR-/HER2-	55(21.2)	245(23.0)	86(18.1)	
Histologic grade				0.044
1	3(1.4)	13(1.3)	5(1.1)	
2	164(74.5)	669(64.8)	298(63.4)	
3	53(24.1)	351(34.0)	167(35.5)	
Nuclear grade				0.155
1	1(0.4)	4(0.4)	1(0.2)	
2	164(71.6)	656(63.2)	299(63.5)	
3	64(27.9)	378(36.4)	171(36.3)	
Lymphovascular invasion				< 0.001
No	203(84.2)	699(67.0)	144(30.5)	
Yes	38(15.8)	344(33.0)	328(69.5)	
Ki-67				< 0.001
< 20	129(60.0)	543(56.3)	204(48.6)	
≥ 20	86(40.0)	421(43.7)	216(51.4)	
Treatment				
Breast surgery type				< 0.001
BCS	131(50.4)	479(45.1)	140(29.5)	
Total mastectomy	129(49.6)	584(54.9)	334(70.5)	
Axillary staging				< 0.001
No axillary surgery	0	2(0.2)	0	
SLNB alone	189(72.7)	628(59.1)	86(18.1)	
ALND ± SLNB	71(27.3)	433(40.7)	388(81.9)	
Adjuvant chemotherapy				< 0.001
No	122(48.6)	658(64.4)	311(67.6)	
Yes	129(51.4)	364(35.6)	149(32.4)	
Radiotherapy				< 0.001
No	60(23.3)	220(20.9)	47(10.0)	
Yes	197(76.7)	834(79.1)	424(90.0)	
Endocrine therapy				0.008
No	90(34.7)	321(30.5)	115(24.4)	
Yes	169(65.3)	732(69.5)	356(75.6)	

Disease-free survival (DFS) was defined as the time from the date of surgery to the occurrence of the first event, including local or regional recurrence, distant metastasis, or death. Overall survival (OS) was defined as the time from the date of surgery to death from any cause. Survival and recurrence data were collected from our institutional breast cancer registry and verified using the national cancer registry database. Kaplan-Meier plots for DFS and OS are shown according to different RCB classes within the four subtypes and according to the subtype within each RCB. The plots were compared using the log-rank test. We estimated the hazard ratio, 95% confidence interval (CI), and effects of clinicopathological variables on recurrence and survival using univariate and multivariate Cox regression analyses. Multicollinearity was assessed using the Variance Inflation Factor (VIF), and all variables included in the multivariate analyses had VIF values below 10, indicating no significant collinearity. To assess the interaction between RCB and HR status as well as HER2 status, interaction terms (*HR × RCB* and *HER2 × RCB*) were incorporated into the Cox regression models.

In Table 4, we used logistic regression analysis to identify factors associated with high RCB (RCB2,3). The model estimated the odds ratios (ORs) with 95% CIs to assess the probability of a patient having high RCB based on clinicopathological factors. All statistical tests were two-sided, with significance threshold set at *p* < 0.05. Statistical analyses were performed using the statistical package for social sciences software (version 21.0; IBM Corp., Armonk, NY, USA).

## Results

### Baseline patient characteristics according to RCB classes

The baseline characteristics of the total study population (*n* = 2,416) are summarized in Supplementary Table 1. The mean age at initial surgery was 48.2 ± 10.0 years, with 44.5% undergoing BCS and 57.7% undergoing sentinel lymph node biopsy (SLNB) alone. (Supplementary Table 1.) The majority of patients (63.2%) were clinical T2 and 45.6% were N1. The HR+/HER2- subtype accounted for 40.9% of the population (Supplementary Table 1).

Excluding RCB0(pCR), the baseline characteristics and treatment parameters of patients with RCB1, 2 and 3 are summarized in Table 1. Clinical factors differed significantly among the RCB classes. RCB3 patients had a relatively higher clinical stage than RCB1 and 2 patients (Table 1). Among RCB3 patients, 40.3% were clinical T3 or 4 compared to 23.4% in RCB1 (*p* < 0.001, Table 1). Among RCB3 patients, HR+/HER2- were the most prevalent subtype (66.0%) and HR-HER2 + were the least common (6.8%) (*p* < 0.001, Table 1). Regarding surgical treatment, 70.5% of RCB3 patients underwent TM and 81.9% underwent axillary lymph node dissection (ALND), while 50.4% of RCB1 patients underwent BCS and 72.7% had SLNB alone (Table 1).

### Disease-free survival according to RCB classes in different subtypes

Overall, DFS and OS progressively declined from RCB0 to RCB3 across all breast cancer subtypes. The OS results are presented in Supplementary (Supplementary Fig. 2a-d). .

**Fig. 1:**
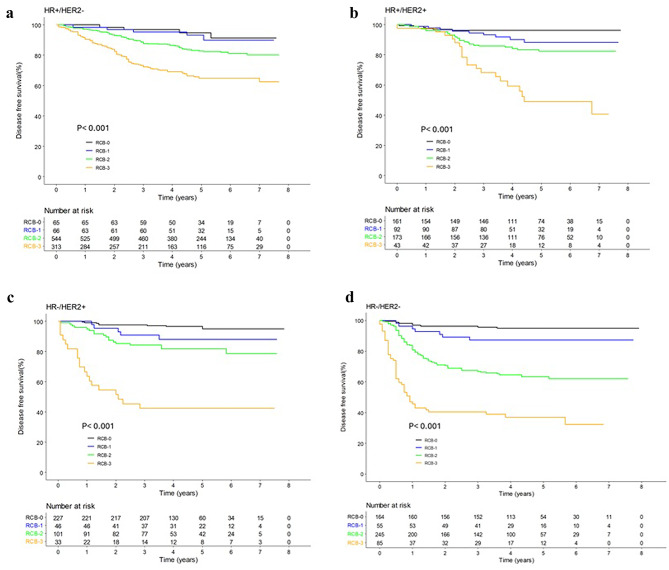
Disease-free survival according to RCB classes and different subtypes. (**a**) HR+/HER2-; (**b**) HR+/HER2+; (**c**) HR-/HER2+; (**d**) HR-/HER2-RCB.

The 5-year DFS of HR+/HER2- patients was 94.8%, 93.3%, 82.7%, and 82.6% for RCB0, RCB1, RCB2, and RCB3, respectively (Fig. 1a). The log-rank test was performed to compare all four Kaplan-Meier curves, and the difference was statistically significant (*p* < 0.001). Among patients with HR + HER2+, it was 96.2%, 82.1%, 82.4%, and 49.0% for RCB0, RCB1, RCB2, and RCB3, respectively (*p* < 0.001) (Fig. 1b). The 5-year DFS among patients with HR-HER2 + was 97.3%, 88.1%, 81.7%, and 40.6% for RCB0, RCB1, RCB2, and RCB3, respectively(*p* < 0.001) (Fig. 1c). In HR+/HER2 + and HR-/HER2 + patients, DFS declined dramatically in RCB3. Among patients with HR-/HER2-, the 5-year DFS declined markedly from RCB2 onward (95.0%, 87.2%, 63.2%, and 37.8% for RCB0, RCB1, RCB2, and RCB3, respectively; *p* < 0.001) (Fig. 1d).

### Disease-free survival across the different subtypes within each RCB class

**Fig. 2:**
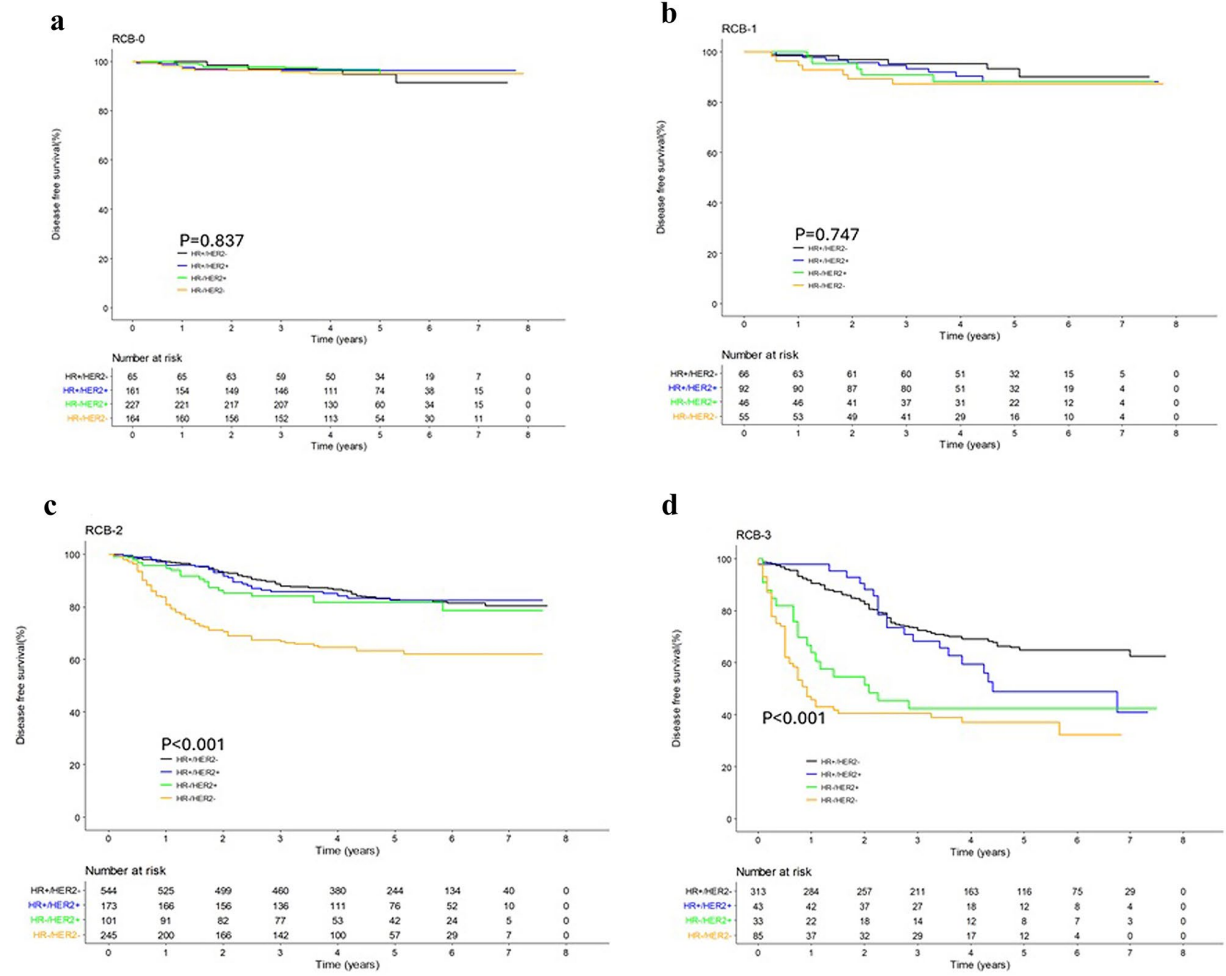
Disease-free survival across the different subtypes within each RCB classes. (**a**) RCB0; (**b**) RCB1; (**c**) RCB3; (**d**) RCB4.

When the DFS of patients between different breast cancer subtypes were compared within each RCB class, no clinically significant differences were observed in the RCB0 and RCB1 classes (*p* = 0.837, *p* = 0.747, respectively) (Fig. 2a, b). In the RCB2 class, DFS was notably lower in HR-/HER2- patients (63.2%) compared to HR+/HER2-, HR+/HER2+, and HR-/HER2 + patients, whose 5-year DFS remained at 82.7%, 82.4%, and 81.7%, respectively (*p* < 0.001, Fig. 2c). In the RCB3 class, while HR-/HER2- patients had the worst DFS (37.8%), HR-/HER2 + patients also showed a markedly poor prognosis, with a 5-year DFS of 40.6%. In contrast, HR+/HER2- and HR+/HER2 + patients had relatively better DFS at 64.8% and 49.0%, respectively (*p* < 0.001, Fig. 2d).

The difference in OS according to the breast subtypes in each RCB class showed a similar trend as DFS (Supplementary Fig. 3a-d).

### Association between clinicopathological parameters, including RCB classes with overall survival

In the univariate Cox regression analysis, patients who underwent TM compared with those who underwent BCS (HR = 1.848, 95% CI: 1.435–2.379, *p* < 0.001), patients who underwent ALND compared with those who underwent SLNB only (HR = 3.101, 95% CI: 2.425–3.965, *p* < 0.001), and patients with higher histologic grade (HR = 2.739 95% CI: 2.425–3.965, *p* < 0.001), nuclear grade (HR = 2.706, 95% CI: 2.126–3.446), LVI (HR = 2.280, 95% CI: 1.792–2.901, *p* < 0.001), and higher Ki-67 proliferation index (HR = 4.219, 95% CI: 3.276–5.434, *p* < 0.001) had higher risk of death (Table 2). HR positivity compared to negativity (HR = 0.453, 95% CI: 0.359–0.573, *p* < 0.001) and HER2 positivity compared to negativity (HR = 0.484, 95% CI: 0.366–0.640, *p* < 0.001) were associated with a lower risk of death (Table 2). Patients with RCB2 and RCB3 had higher risk of death than those with pCR (HR = 4.366, 95% CI: 2.634–7.237, *p* < 0.001, HR = 10.205, 95% CI: 6.149–16.936, *p* < 0.001) (Table 2). The interaction between HR status and RCB classes was statistically significant (*p* = 0.032) whereas the interaction between HER2 status and RCB was not (*p* = 0.107) (Table 2).

**Table 2:** Association between clinicopathological parameters, including RCB classes with overall survival by univariate and multivariate analysis.

Parameter		Univariate			Multivariate		
		HR	95%CI	*p*-value	HR	95%CI	*p*-value
Age	< 50 vs. ≥50 years	0.994	0.786–1.256	0.958			
Operation type	BCS vs. TM	1.848	1.435–2.379	< 0.001	1.421	1.094–1.846	0.009
Axillary Surgery type	SLNB vs. ALND	3.101	2.425–3.965	< 0.001	2.252	1.681–3.018	< 0.001
Hormone receptor	Negative vs. positive	0.453	0.359–0.573	< 0.001			
HER2	Negative vs. positive	0.484	0.366–0.640	< 0.001	0.446	0.329–0.605	< 0.001
RCB				< 0.001			< 0.001
	0 vs. 1	1.802	0.875–3.710	0.110	1.443	0.688–3.026	0.332
	0 vs. 2	4.366	2.634–7.237	< 0.001	2.626	1.527–4.515	0.001
	0 vs. 3	10.205	6.149–16.936	< 0.001	4.118	2.264–7.490	< 0.001
Hormone Receptor x RCB				0.032			0.040
	HR = 1, 0 vs.1	0.796	0.176–3.588	0.766	0.844	0.186–3.836	0.827
	HR = 1, 0 vs. 2	0.477	0.159–1.429	0.186	0.512	0.168–1.562	0.239
	HR = 1, 0 vs. 3	0.285	0.094–0.860	0.026	0.304	0.099–0.928	0.037
HER2 x RCB				0.107			
	HER2 = 1, 0 vs.1	0.879	0.202–3.827	0.864			
	HER2 = 1, 0 vs. 2	0.465	0.155–1.391	0.171			
	HER2 = 1, 0 vs. 3	0.990	0.335–2.923	0.985			
Histologic grade	Low(1,2) vs. high(3)	2.739	2.150–3.491	< 0.001	1.479	1.124–1.945	0.005
Nuclear grade	Low(1,2) vs. high(3)	2.706	2.126–3.446	< 0.001			
Lymphovascular invasion	No vs. Yes	2.280	1.792–2.901	< 0.001	1.582	1.196–2.094	0.001
Ki-67	Low(< 20) vs. high(20≤)	4.219	3.276–5.434	< 0.001	2.170	1.586–2.970	< 0.001
Radiotherapy	No vs. Yes	1.454	1.044–2.024	0.027			
Endocrine therapy	No vs. Yes	0.452	0.356–0.571	< 0.001			
Adjuvant chemotherapy	No vs. Yes	0.832	0.653–1.060	0.136			

RCB, residual cancer burden; HER2, human epidermal growth factor receptor 2; HR, hazard ratio; BCS, breast-conserving surgery; TM, tumor mastectomy; SLNB, sentinel lymph node biopsy; ALND, axillary lymph node dissection;

In the multivariate analysis, most clinical factors remained statistically significant, including surgery type, HER2 status, RCB, tumor grade, LVI and Ki-67 proliferation index (Table 2).

### Association between clinicopathological parameters, including RCB classes with disease-free survival

In the univariate Cox regression analysis with respect to DFS, younger age (< 50 vs. ≥ 50 years HR = 0.821, 95% CI: 0.682–0.989, *p* < 0.038), TM, (HR = 1.573, 95% CI: 1.300–1.903, *p* < 0.001), ALND (HR = 2.411, 95% CI: 2.003–2.901, *p* < 0.001), higher histologic grade (HR = 1.994, 95% CI: 1.651–2.407, *p* < 0.001) and nuclear grade (HR = 1.946, 95% CI: 1.615–2.344, *p* < 0.001), LVI (HR = 2.601, 95% CI: 2.158–3.138, *p* < 0.001), and higher Ki-67 proliferation index (HR = 3.337, 95% CI: 2.756–4.046, *p* < 0.001) were associated with a higher risk of recurrence (Table 3). Patients with HR and HER2 receptor had less risk of recurrence (HR- vs. HR + HR = 0.764, 95% CI: 0.636–0.917, *p* = 0.004, HER2- vs. HER2 + HR = 0.533, 95% CI: 0.431–0.659, *p* < 0.001) (Table 3). Compared with patients with RCB0, those with RCB1, RCB2, and RCB3 had a higher risk of recurrence (RCB0 vs. RCB1 HR = 2.436, 95% CI: 1.415–4.196, *p* = 0.001, RCB0 vs. RCB2 HR = 5.321, 95% CI: 3.545–7.989, *p* < 0.001 RCB0 vs. RCB3 HR = 12.155, 95% CI: 8.071–18.306, *p* < 0.001) (Table 3). The interaction between HR status and RCB classes was statistically significant (*p* = 0.032) whereas the interaction between HER2 status and RCB was not (*p* = 0.072) (Table 3).

**Table 3:** Association between clinicopathological parameters, including RCB classes with disease-free survival by univariate and multivariate analysis.

Parameter		Univariate			Multivariate		
		HR	95%CI	*p*-value	HR	95%CI	*p*-value
Age	< 50 vs. ≥50 years	0.821	0.682–0.989	0.038	0.751	0.617–0.914	0.043
Operation type	BCS vs. TM	1.573	1.300–1.903	< 0.001			
Axillary Surgery type	SLNB vs. ALND	2.411	2.003–2.901	< 0.001	1.562	1.249–1.953	< 0.001
Hormone receptor	Negative vs. positive	0.764	0.636–0.917	0.004			
HER2	Negative vs. positive	0.533	0.431–0.659	< 0.001	0.692	0.542–0.883	0.003
RCB				< 0.001			< 0.001
	0 vs. 1	2.436	1.415–4.196	0.001	1.895	1.077–3.333	0.027
	0 vs. 2	5.321	3.545–7.989	< 0.001	3.436	2.209–5.344	< 0.001
	0 vs. 3	12.155	8.071–18.306	< 0.001	6.095	3.723–9.979	< 0.001
Hormone receptor x RCB				0.032			0.079
	HR = 1, 0 vs.1	0.683	0.227–2.054	0.498	0.690	0.227–2.095	0.512
	HR = 1, 0 vs. 2	0.423	0.184–0.974	0.043	0.487	0.211–1.127	0.093
	HR = 1, 0 vs. 3	0.320	0.136–0.754	0.009	0.363	0.154–0.857	0.021
HER2 x RCB				0.071			
	HER2 = 1, 0 vs.1	1.455	0.491–4.306	0.498			
	HER2 = 1, 0 vs. 2	1.048	0.454–2.415	0.913			
	HER2 = 1, 0 vs. 3	1.915	0.824–4.451	0.131			
Histologic grade	Low(1,2) vs. high(3)	1.994	1.651–2.407	< 0.001	1.331	1.058–1.674	0.015
Nuclear grade	Low(1,2) vs. high(3)	1.946	1.615–2.344	< 0.001			
Lymphovascular invasion	No vs. Yes	2.601	2.158–3.138	< 0.001	1.796	1.445–2.233	< 0.001
Ki-67	Low(< 20) vs. high(20≤)	3.337	2.756–4.046	< 0.001	2.040	1.612–2.582	< 0.001
Radiotherapy	No vs. Yes	1.230	0.964–1.571	0.096			
Endocrine therapy	No vs. Yes	0.748	0.622–0.900	0.002			
Adjuvant chemotherapy	No vs. Yes	0.890	0.737–1.074	0.224			

RCB, residual cancer burden; HER2, human epidermal growth factor receptor 2; HR, hazard ratio; BCS, breast-conserving surgery; TM, tumor mastectomy; SLNB, sentinel lymph node biopsy; ALND, axillary lymph node dissection.

In the multivariate analysis, the following parameters were significantly associated with a higher risk of recurrence, which was consistent with the univariate analysis results: younger age, ALND, HER2 negativity, higher RCB, higher histologic grade, LVI and higher Ki-67 proliferation index (Table 3).

### Association between clinicopathological factors and high RCB(RCB2,3) by univariate and multivariate analysis

In the univariate analysis, younger patients were associated with higher RCB classes (50 < vs. ≥50 years OR = 0.763, 95% CI: 0.645–0.901, *p* = 0.002) (Table 4). Undergoing TM rather than BCS (OR = 1.591, 95% CI: 1.347–1.81, *p* < 0.001) and undergoing ALND rather than SLNB (OR = 3.955, 95% CI: 3.219–4.770, *p* < 0.001), higher clinical stage (T1 vs. T4 OR = 2.065, 95% CI: 1.301–3.277, *p* = 0.002, N0 vs. N3 OR = 1.412, 95% CI: 1.103–1.807, *p* = 0.006), LVI (OR = 10.129, 95% CI: 7.255–14.141, *p* < 0.001), higher Ki-67 proliferation index (OR = 3.629, 95% CI: 2.504–3.534, *p* < 0.001) were associated with having high RCB(RCB2,3) (Table 4). HR+/HER2- patients had the highest odds of having higher RCB (RCB2,3) compared to other subtypes. This trend remained significant in the multivariate analysis, where HR+/HER2- continued to show the strongest association with high RCB (Table 4).

**Table 4:** Association between clinicopathological factors and high RCB (RCB2,3) by univariate and multivariate analysis.

Parameter		Univariate			Multivariate		
		OR	95%CI	*p*-value	OR	95%CI	*p*-value
Age	50 < vs. ≥50	0.763	0.645–0.901	0.002			
Operation type	BCS vs. TM	1.591	1.347–1.881	< 0.001	2.731	2.148–3.473	< 0.001
Axillary Surgery type	SLNB vs. ALND	3.955	3.279–4.770	< 0.001			
Clinical T Stage				0.004			
	T1 vs. 2	1.179	0.872–1.595	0.285			
	T1 vs. 3	1.471	1.051–2.059	0.024			
	T1 vs. 4	2.065	1.301–3.277	0.002			
Clinical N stage				0.008			
	N0 vs. N1	1.379	1.131–1.683	0.002			
	N0 vs. N2	1.222	0.877–1.703	0.235			
	N0 vs. N3	1.412	1.103–1.807	0.006			
	M0 vs. M1	1.970	1.215–3.195	0.006			
Histologic grade	Low(1,2) vs. high(3)	1.191	0.946–1.500	0.137			
Nuclear grade	Low(1,2) vs. high(3)	1.089	0.886–1.337	0.418			
Lymphovascular invasion	No vs. Yes	10.129	7.255–14.141	< 0.001	4.871	3.483–6.813	< 0.001
Ki-67	Low(< 20) vs. high(20≤)	3.629	2.900–4.543	< 0.001	6.424	4.883–8.452	< 0.001
Subtype				< 0.001			< 0.001
	HR+/HER2- vs.HR+/HER2+	0.131	0.101–0.169	< 0.001	0.110	0.082–0.149	< 0.001
	HR+/HER2- vs. HR-/HER2	0.075	0.057–0.099	< 0.001	0.044	0.030–0.063	< 0.001
	HR+/HER2- vs. HR-/HER2	0.230	0.179–0.296	< 0.001	0.168	0.124–0.227	< 0.001

RCB, residual cancer burden; OR, odds ratio; HER2, human epidermal growth factor receptor 2; HR, hormone receptor; BCS, breast-conserving surgery; TM, tumor mastectomy; SLNB, sentinel lymph node biopsy; ALND, axillary lymph node dissection.

## Discussion

In this study, we assessed the impact of RCB on survival in different breast cancer subtypes and explored the risk factors of high RCB. Our findings show that patients with RCB2 and RCB3 with the HR-/HER2- subtype and those with RCB3 with the HR-/HER2 + subtype experienced particularly unfavorable prognoses despite receiving standard neoadjuvant and adjuvant therapy. The risk of recurrence increases as the extent of RD increases.

Previous studies have validated the prognostic value of RCB and reported that the measurements of RCB were highly reproducible by different pathologists^14,15^. However, its clinical implementation remains inconsistent due to variations in reporting practices. A survey of 26 breast pathologists in academic centers across the United States found that while 74% report RCB or provide sufficient information for its calculation, 26% do not, highlighting discrepancies in its use^10^. The technical challenges in specimen handling and the lack of consensus on its necessity further limits its integration into routine clinical practice^11,16^. In our study, the risk of recurrence increased at different scales according to the breast cancer subtypes, suggesting that incorporating both RCB and subtype information enable more detailed risk assessment and more precise adjuvant treatment decisions. Given these findings, we aim to further emphasize the clinical significance of RCB and advocate for its standardized integration into routine practice.

For HR+/HER2- advanced breast cancer, CDK4/6 inhibitors are increasingly utilized with supporting evidence from trials like monarchE. While trials such as monarchE, NATALEE, and PALLAS included patients who had received NAC and may have had residual disease, the efficacy of CDK4/6 inhibitors in this subgroup remains unclear, as outcomes have not been specifically addressed^17–19^. The PENELOPE-B trial enrolled post-NAC patients with a clinical pathological staging-estrogen receptor grading(CPS-EG) score ≥ 3 or 2 and ypN + but it failed to demonstrate efficacy of one-year palbociclib compared to the placebo^20^. Given that HR+/HER2- patients tend to exhibit RCB2 or RCB3 rather than RCB1 or pCR in our study, the role of CDK4/6 inhibitors in this high residual disease population needs to be specifically addressed in future studies.

Previous studies have shown a weaker association between RCB and survival in HR+/HER2- patients compared to other subtypes^7^. Similarly, when we performed Cox regression analysis within HR+/HER2- group, the increase in hazard ratio by RCB1 (relative to pCR) was not significant compared to RCB2 and 3. Notably, the interaction between HR status and RCB classes was statistically significant for both DFS and OS in our study. This weaker association may be attributed to factors such as heterogeneous HR classification, early recurrences in RCB0 patients treated with bevacizumab, suboptimal specimen sampling, and the greater prognostic influence of long-term endocrine therapy rather than chemotherapy response in HR+/HER2 − tumors^7^. Additionally, HR + tumors tend to shrink discordantly with imaging techniques such as magnetic resonance imaging^21,22^. However, as this study was conducted in a single center, clinical practices—including surgical procedures, imaging interpretation, and pathology specimen handling—were relatively standardized, enhancing data consistency and accuracy.

In a study assessing RCB and EFS using data from the I-SPY2 trial, patients with HER2 + cancers had higher rates of pCR, consistent with our study’s finding that the HR-/HER2 + subtype had the largest number of patients with pCR^23^. In the previous study, RCB was a prognostic factor for all subtypes^7^. Among patients with HR-/HER2+, 3-year EFS was 50% for those with RCB2 and 33% for those with RCB3. Our study showed better survival rates in patients with RCB2 and HR-/HER2+, with 3-year DFS of 84.3% for RCB2 and 40.3% for RCB3. The Kaplan-Meier curve in the previous study showed that RCB2 separated from RCB1 after 2 years and declined sharply^7^. In contrast, in our study, the RCB2 curve was closer to RCB1 and demonstrated a much better prognosis than RCB3. Our study included a higher proportion of cT1 patients (8% vs. 3%); however, the different patterns of the RCB Kaplan-Meier curve could be explained by the difference in NAC regimens. Considering our study’s timing, our patients were treated more recently, and most of them received the Docetaxel-cyclophosphamide-trastuzumab-pertuzumab regimen for NAC, while the patients in the I-SPY2 trial received the docetaxel-trastuzumab-pertuzumab-doxorubicin-cyclophosphamide protocol. In our study, patients with HR-/HER2 + and RCB3 had a poor prognosis. The KATHERINE trial results demonstrated the survival benefit of trastuzumab-emtansine over trastuzumab, suggesting potential benefits for the HER2 + patients who are non-responsive to NAC^24^.

In an exploratory analysis using KEYNOTE-522 trial, the distribution of RCB was evaluated according to the treatment group. Adding pembrolizumab improved the pCR rates and resulted in a shift of patients to lower RCB classes across the entire spectrum when compared to the placebo^13^. The pCR rate was 56.2% in the placebo + chemo arm and 63.4% in the permbro + chemo arm in this previous study compared with the 29.8% (164/550) for patients with HR-/HER2- in our study^13^. This could be due to the difference in NAC regimens and the inclusion of N3 patients in our study, accounting for 19.3% of the total patients. The KEYNOTE-522 trial enrolled patients with tumor stage T1c and nodal stage N1-2 as well as those with tumor stage T2-4 and nodal stage N0-2, according to the criteria set by the AJCC, 7th edition^13^. Notably, patients with RCB2 benefited the most from pembrolizumab in the study^13^. RCB3 patients with HR-/HER2- subtype remained at high risk despite pembrolizumab treatment highlighting the need for intensive post-treatment surveillance or tailored therapeutic strategies beyond standard adjuvant therapy.

HR+/HER2- cancers show poor response to chemotherapy and achieve lower pCR rates^25^. The HR+/HER2- subtype is one of the factors associated with a higher RCB. Other factors include TM, presence of LVI, and high Ki-67 proliferation index. A high Ki-67 proliferation index has been suggested to be a predictor of good response to NAC in some studies^26,27^. However, in other studies with contrasting results, the cutoff value of the Ki-67 index was highly variable from 5 to 35%^28,29^. Pre-therapeutic values have been used to validate the predictive value of Ki-67, whereas in our study, we used post-treatment values due to data accessibility. Our study proved the prognostic effect of Ki-67; patients with a high proliferation index were more likely to have recurrence or death according to the univariate and multivariate analyses, including RCB, which was consistent with other studies.

This study has some limitations, including its retrospective design, which may introduce selection bias and limit its generalizability. Moreover, the follow-up period was relatively short with the median follow-up period of 57 months, therefore including recently treated patients. Additionally, the exact cause of death could not be determined. However, survival status was systematically tracked using the National Health Insurance System, where loss of insurance eligibility serves as a reliable indicator of death. Despite these limitations, the study is strengthened by the availability of information on adjuvant radiotherapy and ET, as well as a large sample size from a single center, which ensures robust statistical power. This study proved that the RCB is a highly significant prognostic marker. However, measuring the RCB is not yet a routine procedure nationally or globally, making our data particularly valuable. Future research should focus on prospective studies that tailor adjuvant therapy based on an individual’s RCB class following NAC. This approach could help determine whether unnecessary adjuvant treatments can be minimized while intensifying systemic therapy for patients who require additional intervention.

## Conclusion

Our study elaborates on how the risk of recurrence and death increases with the RCB classes, with the impact differing across breast cancer subtypes. Patients with RCB2 and RCB3 with HR-/HER2- subtypes and those with RCB3 with HR-/HER2 + subtypes experienced poor prognoses despite receiving standard neoadjuvant and adjuvant therapy. To improve survival outcomes of those patients, RCB can serve as a key factor in treatment decisions, guiding the need for intensified adjuvant therapy and closer surveillance, particularly in conjunction with breast cancer subtypes.

## Electronic supplementary material

Below is the link to the electronic supplementary material.

Supplementary Material 1

Supplementary Material 2

Supplementary Material 3

Supplementary Material 4

Supplementary Material 5

Supplementary Material 6

Supplementary Material 7

Supplementary Material 8

Supplementary Material 9

Supplementary Material 10

## Data Availability

The datasets analyzed during the current study are not publicly available due to the protection of the personal information of patients but are available from the corresponding author on reasonable request.

## Abbreviations

AJCC: American Joint Committee on Cancer
ALND: Axillary lymph node dissection
BCS: Breast-conserving surgery
CTx: Chemotherapy
DFS: Disease-free survival
EFS: Event-free survival
ET: Endocrine therapy
HER2: Human epidermal growth factor receptor 2
HR: Hormone receptor
LVI: Lymphovascular invasion
OS: Overall survival
pCR: Pathologic complete response
RD: Residual disease
SLNB: Sentinel lymph node biopsy
TM: Tumor mastectomy
TNBC: Triple-negative breast cancer

## References

[1] Mieog, J. S., van der Hage, J. A. & van de Velde, C. J. Neoadjuvant chemotherapy for operable breast cancer. *Br. J. Surg.* **94** (10), 1189–1200. 10.1002/bjs.5894 (2007). 17701939 10.1002/bjs.5894

[2] Cortazar, P. et al. Pathological complete response and long-term clinical benefit in breast cancer: the CTNeoBC pooled analysis. *Lancet* **384** (9938), 164–172. 10.1016/S0140-6736(13)62422-8 (2014). 24529560 10.1016/S0140-6736(13)62422-8

[3] Fisher, B. et al. Effect of preoperative chemotherapy on the outcome of women with operable breast cancer. *J. Clin. Oncol.* **16** (8), 2672–2685. 10.1200/JCO.1998.16.8.2672 (1998). 9704717 10.1200/JCO.1998.16.8.2672

[4] Spring, L. et al. Pathologic complete response after neoadjuvant chemotherapy and long-term outcomes among young women with breast cancer. *J. Natl. Compr. Canc Netw.* **15** (10), 1216–1223. 10.6004/jnccn.2017.0158 (2017). 28982747 10.6004/jnccn.2017.0158

[5] Prowell, T. M. & Pazdur, R. Pathological complete response and accelerated drug approval in early breast cancer. *N Engl. J. Med.* **366** (26), 2438–2441. 10.1056/NEJMp1205737 (2012). 22646508 10.1056/NEJMp1205737

[6] Symmans, W. F. et al. Measu rement of residual breast cancer burden to predict survival after neoadjuvant chemotherapy. *J. Clin. Oncol.* **25** (28), 4414–4422. 10.1200/JCO.2007.10.6823 (2007). 17785706 10.1200/JCO.2007.10.6823

[7] Yau, C. et al. Residual cancer burden after neoadjuvant chemotherapy and long-term survival outcomes in breast cancer: a multicentre pooled analysis of 5161 patients. *Lancet Oncol.* **23** (1), 149–160. 10.1016/S1470-2045(21)00589-1 (2022). 34902335 10.1016/S1470-2045(21)00589-1 PMC9455620

[8] Amin, M. B. et al. **AJCC Cancer Staging Manual** 8th edn (Springer, 2017).

[9] National Comprehensive Cancer Network. NCCN Clinical Practice Guidelines in Oncology: Breast Cancer. Version 4.2023. Published September 8. Accessed March 5, 2025. (2023). Available at: https://www.nccn.org

[10] Lanjewar, S., Patil, P. & Fineberg, S. Pathologic reporting practices for breast cancer specimens after neoadjuvant chemotherapy—a survey of pathologists in academic institutions across the united States. *Mod. Pathol. Volume* **33**, Issue 1,2020,Pages 91–98,ISSN 0893–3952, 10.1038/s41379-019-0326-5 10.1038/s41379-019-0326-5 31383962

[11] Park, C. K., Jung, W. H. & Koo, J. S. Pathologic evaluation of breast Cancer after neoadjuvant therapy. *J. Pathol. Transl Med.* **50** (3), 173–180. 10.4132/jptm.2016.02.02 (2016). Epub 2016 Apr 11. PMID: 27068026; PMCID: PMC4876080. 27068026 10.4132/jptm.2016.02.02 PMC4876080

[12] Yin, L., Duan, J. J., Bian, X. W. & Yu, S. C. Triple-negative breast cancer molecular subtyping and treatment progress. *Breast Cancer Res.* **22** (1), 61. 10.1186/s13058-020-01296-5 (2020). 32517735 10.1186/s13058-020-01296-5 PMC7285581

[13] Pusztai, L. et al. Event-free survival by residual cancer burden with pembrolizumab in early-stage TNBC: exploratory analysis from KEYNOTE-522. *Ann. Oncol.* **35** (5), 429–436. 10.1016/j.annonc.2024.02.002 (2024). 38369015 10.1016/j.annonc.2024.02.002

[14] Peintinger, F. et al. Reproducibility of residual cancer burden for prognostic assessment of breast cancer after neoadjuvant chemotherapy. *Mod. Pathol.* **28** (7), 913–920. 10.1038/modpathol.2015.53 (2015). 25932963 10.1038/modpathol.2015.53 PMC4830087

[15] Naidoo, K., Parham, D. M. & Pinder, S. E. An audit of residual cancer burden reproducibility in a UK context. *Histopathology* **70** (2), 217–222. 10.1111/his.13054 (2017). 27496095 10.1111/his.13054

[16] Sahoo, S. et al. Standardizing pathologic evaluation of breast carcinoma after neoadjuvant chemotherapy. *Arch. Pathol. Lab. Med.* **147** (5), 591–603. 10.5858/arpa.2022-0021-EP (2022). 35976643 10.5858/arpa.2022-0021-EP

[17] Johnston, S. R. D. et al. Abemaciclib plus endocrine therapy for hormone receptor-positive, HER2-negative, node-positive, high-risk early breast cancer (monarchE): results from a Preplanned interim analysis of a randomised, open-label, phase 3 trial. *Lancet Oncol.* **24** (1), 77–90 (2023). 36493792 10.1016/S1470-2045(22)00694-5 PMC11200328

[18] Slamon, D. et al. Ribociclib plus endocrine therapy in early breast cancer. *N. Engl. J. Med.* **March 20**10.1056/NEJMoa2309504 (2024). 10.1056/NEJMc2404917 38899707

[19] Gnant, M. et al. Adjuvant Palbociclib for early breast cancer: the PALLAS trial results (ABCSG-42/AFT-05/BIG-14-03). *J. Clin. Oncology: Official J. Am. Soc. Clin. Oncol.* **40** (3), 282–293. 10.1200/JCO.21.02554 (2022). 10.1200/JCO.21.02554 PMC10476784 34874182

[20] Loibl, S. et al. Palbociclib for residual High-Risk invasive HR-Positive and HER2-Negative early breast Cancer-The Penelope-B trial. *J. Clin. Oncology: Official J. Am. Soc. Clin. Oncol.* **39** (14), 1518–1530. 10.1200/JCO.20.03639 (2021). 10.1200/JCO.20.03639 33793299

[21] Zheng, C. H. et al. Meta-analysis of shrinkage mode after neoadjuvant chemotherapy for breast cancers: association with hormonal receptor. *Front. Oncol.* **11**, 617167. 10.3389/fonc.2021.617167 (2021). 35444932 10.3389/fonc.2021.617167 PMC9014257

[22] Mukhtar, R. A. et al. TRIAL and ACRIN 6657 Clinically meaningful tumor reduction rates vary by prechemotherapy MRI phenotype and tumor subtype in the I-SPY 1 TRIAL (CALGB 150007/150012; ACRIN 6657). Ann Surg Oncol 20(12):3823–3830. (2013). 10.1245/s10434-013-3038-y 10.1245/s10434-013-3038-y PMC3824937 23780381

[23] Symmans, W. F. et al. Assessment of residual cancer burden and event-free survival in neoadjuvant treatment for high-risk breast cancer: an analysis of data from the I-SPY2 randomized clinical trial. *JAMA Oncol.* **7** (11), 1654–1663. 10.1001/jamaoncol.2021.3690 (2021). 34529000 10.1001/jamaoncol.2021.3690 PMC8446908

[24] von Minckwitz, G. et al. Trastuzumab emtansine for residual invasive HER2-positive breast cancer. *N Engl. J. Med.* **380** (7), 617–628. 10.1056/NEJMoa1814017 (2019). 30516102 10.1056/NEJMoa1814017

[25] von Minckwitz, G. et al. Definition and impact of pathologic complete response on prognosis after neoadjuvant chemotherapy in various intrinsic breast cancer subtypes. *J. Clin. Oncol.* **30** (15), 1796–1804. 10.1200/JCO.2011.38.8595 (2012). 22508812 10.1200/JCO.2011.38.8595

[26] Sueta, A. et al. Clinical significance of pretherapeutic Ki67 as a predictive parameter for response to neoadjuvant chemotherapy in breast cancer: is it equally useful across tumor. *subtypes? Surg.* **155** (5), 927–935. 10.1016/j.surg.2014.01.009 (2014). 10.1016/j.surg.2014.01.009 24582496

[27] Tao, M., Chen, S., Zhang, X. & Zhou, Q. Ki-67 labeling index is a predictive marker for a pathological complete response to neoadjuvant chemotherapy in breast cancer: A meta-analysis. *Med. (Baltim)*. **96** (51), e9384. 10.1097/MD.0000000000009384 (2017). 10.1097/MD.0000000000009384 PMC5758242 29390540

[28] Jones, R. L. et al. Relationship between oestrogen receptor status and proliferation in predicting response and long-term outcome to neoadjuvant chemotherapy for breast cancer. *Breast Cancer Res. Treat.* **119** (2), 315–323. 10.1007/s10549-009-0329-x (2010). 19247830 10.1007/s10549-009-0329-x

[29] Tordai, A. et al. Evaluation of biological pathways involved in chemotherapy response in breast cancer. *Breast Cancer Res. PMC*. **2 10** (2), R37 (2008). 10.1186/bcr2088 PMC2397539 18445275

