# Supplementary material for: Prognostic value of residual cancer burden after neoadjuvant chemotherapy in breast cancer: a comprehensive subtype-specific analysis
Source: Sci Rep. 2025 Apr 22;15:13977. doi: 10.1038/s41598-025-98176-9 (PMC12015579; doi:10.1038/s41598-025-98176-9)
Supplement: Supplementary file 1 — Supplementary Material 1 [file 41598_2025_98176_MOESM1_ESM.docx]

**Supplementary Fig1, RCB formula**

**RCB = 1.4 (f_inv_ d_prim_) ^0.17^ + [4(1 -0.75^LN^)d_met_] ^0.17^**

d_prim_ = √d_1_d_2_

f_inv_= [1-(%CIS/100)] x (%CA/100)

d_prim_ : Primary tumor bed dimensions

d_met_ : Size of metastasis

f_inv_ : Cellularity fraction of invasive cancer

CIS, carcinomain situ; CA, carcinoma

**Supplementary Table1.** **Baseline patient characteristics according to RCB classes (n=2,416)**

| **Characteristics** | Total  (n=2,416)  N (%) | RCB=0  (N=619) (25.6%) | RCB=1  (N=260) (10.8%) | RCB=2  (N=1063)  (44.0) | RCB=3  (N=474)  (19.6) | p-value |
| --- | --- | --- | --- | --- | --- | --- |
| **Age at initial surgery (years), mean ± SD** | 48.2±10.0 | 49.2±10.5 | 48.3±9.7 | 47.2±9.8 | 48.9±10.0 | <0.001 |
| **Body mass index (kg/m^2^)** | 23.9±3.9 | 23.7±4.3 | 23.5±3.0 | 23.9±3.8 | 24.6±4.3 | 0.004 |
| **Breast surgery type** |  |  |  |  |  | <0.001 |
| **BCS** | 1,074 (44.5) | 324(52.3) | 131(50.4) | 479(45.1) | 140(29.5) |  |
| **Total mastectomy** | 1,342 (55.5) | 295(47.7) | 129(49.6) | 584(54.9) | 334(70.5) |  |
| **Axillary staging** |  |  |  |  |  | <0.001 |
| **No axillary surgery** | 2(0.1) | 0 | 0 | 2(0.2) | 0 |  |
| **SLNB alone** | 1395(57.7) | 492(79.5) | 189(72.7) | 628(59.1) | 86(18.1) |  |
| **ALND** ± **SLNB** | 1019(42.2) | 127(20.5) | 71(27.3) | 433(40.7) | 388(81.9) |  |
| **Clinical T stage** |  |  |  |  |  | <0.001 |
| **Tis** | 5(0.2) | 3(0.5) | 0 | 2(0.2) | 0 |  |
| **T1** | 196(8.1) | 58(9.4) | 24(9.2) | 87(8.2) | 27(5.7) |  |
| **T2** | 1528(63.2) | 404(65.3) | 175(67.3) | 693(65.2) | 256(54.0) |  |
| **T3** | 536(22.2) | 126(20.4) | 50(19.2) | 227(21.4) | 133(28.1) |  |
| **T4** | 151(6.3) | 28(4.5) | 11(4.2) | 54(5.1) | 58(12.2) |  |
| **Clinical N stage** |  |  |  |  |  | <0.001 |
| **N0** | 5(0.2) | 3(0.5) | 0 | 2(0.2) | 0 |  |
| **N1** | 1102(45.6) | 260(42.0) | 117(45.0) | 479(45.1) | 246(51.9) |  |
| **N2** | 192(7.9) | 52(8.4) | 19(7.3) | 81(7.3) | 40(8.4) |  |
| **N3** | 466(19.3) | 114(18.4) | 43(16.5) | 174(16.4) | 135(28.5) |  |
| **Clinical M stage** |  |  |  |  |  | 0.004 |
| **M0** | 2320(96.0) | 602(97.3) | 255(92.1) | 1020(96.0) | 443(93.5) |  |
| **M1** | 96(4.0) | 17(2.7) | 5(1.9) | 43(4.0) | 31(6.5) |  |
| **Total number of lymph nodes harvested, mean ± SD** | 10.0±7.5 | 7.7±5.6 | 7.8±5.5 | 9.4±6.5 | 15.5±9.6 | <0.001 |
| **Number of positive lymph nodes, mean ± SD** | 1.9 ±4.3 | 0.0±0.2 | 0.3±0.6 | 1.5±2.3 | 6.2±7.5 | <0.001 |
| **Histologic grade** |  |  |  |  |  | 0.038 |
| **1** | 21(1.1) | 0 | 3(1.4) | 13(1.3) | 5(1.1) |  |
| **2** | 1263(65.3) | 132(62.6) | 164(74.5) | 669(64.8) | 298(63.4) |  |
| **3** | 649(33.6) | 79(37.4) | 53(24.1) | 351(34.0) | 167(35.5) |  |
| **Unknown** | 482 |  |  |  |  |  |
| **Nuclear grade** |  |  |  |  |  | 0.003 |
| **1** | 12(0.6) | 6(1.9) | 1(0.4) | 4(0.4) | 1(0.2) |  |
| **2** | 1304(63.5) | 185(58.9) | 164(71.6) | 656(63.2) | 299(63.5) |  |
| **3** | 736(35.9) | 123(39.2) | 64(27.9) | 378(36.4) | 171(36.3) |  |
| **Unknown** | 364 |  |  |  |  |  |
| **Lymphovascular invasion** |  |  |  |  |  | <0.001 |
| **No** | 1364(65.7) | 318(99.1) | 203(84.2) | 699(67.0) | 144(30.5) |  |
| **Yes** | 713(34.3) | 3(0.9) | 38(15.8) | 344(33.0) | 328(69.5) |  |
| **Unknown** | 339 |  |  |  |  |  |
| **Hormone receptor** |  |  |  |  |  | <0.001 |
| **Negative** | 958(39.7) | 392(63.4) | 102(39.2) | 346(32.5) | 118(24.9) |  |
| **Positive** | 1457(60.3) | 226(36.6) | 158(60.8) | 717(67.5) | 356(75.1) |  |
| **Unknown** | 1 |  |  |  |  |  |
| **HER2** |  |  |  |  |  | <0.001 |
| **Negative** | 1538(63.7) | 229(37.1) | 121)46.7) | 789(74.2) | 399(84.2) |  |
| **Positive** | 876(36.3) | 389(62.9) | 138(53.3) | 274(25.8) | 75(15.8) |  |
| **Unknown** | 2 |  |  |  |  |  |
| **Ki-67** |  |  |  |  |  | <0.001 |
| **<20** | 1262(62.5) | 386(91.7) | 129(60.0) | 543(56.3) | 204(48.6) |  |
| **≥20** | 758(37.5) | 35(8.3) | 86(40.0) | 421(43.7) | 216(51.4) |  |
| **Unknown** | 396 |  |  |  |  |  |
| **Subtype** |  |  |  |  |  | <0.001 |
| **HR+/HER2-** | 988(40.9) | 65(10.5) | 66(25.5) | 544(51.2) | 313(66.0) |  |
| **HR+/HER2+** | 469(19.4) | 161(26.1) | 92(65.5) | 173(16.3) | 43(9.1) |  |
| **HR-/HER2+** | 406(16.8) | 227(36.8) | 46(17.8) | 101(9.5) | 32(6.8) |  |
| **HR-/HER2-** | 550(22.8) | 164(26.6) | 55(21.2) | 245(23.0) | 86(18.1) |  |
| **Unknown** | 3 |  |  |  |  |  |
| **Adjuvant chemotherapy** |  |  |  |  |  | <0.001 |
| **No** | 1368(58.4) | 277(45.6) | 122(48.6) | 658(64.4) | 311(67.6) |  |
| **Yes** | 973(41.6) | 331(54.4) | 129(51.4) | 364(35.6) | 149(32.4) |  |
| **Unknown** | 75 |  |  |  |  |  |
| **Radiotherapy** |  |  |  |  |  | <0.001 |
| **No** | 455(19.0) | 128(20.9) | 60(23.3) | 220(20.9) | 47(10.0) |  |
| **Yes** | 1940(81.0) | 485(79.1) | 197(76.7) | 834(79.1) | 424(90.0) |  |
| **Unknown** | 21 |  |  |  |  |  |
| **Endocrine therapy** |  |  |  |  |  | <0.001 |
| **No** | 908(37.9) | 382(62.2) | 90(34.7) | 321(30.5) | 115(24.4) |  |
| **Yes** | 1489(62.1) | 232(37.8) | 169(65.3) | 732(69.5) | 356(75.6) |  |
| **Unknown** | 19 |  |  |  |  |  |

**Supplementary Table2. Distribution of neoadjuvant chemotherapy and anti-HER2 therapy according to the breast cancer subtypes (n=2,415)**

|  | HR+/HER2-  N (%) | HR+/HER2+  N (%) | HR-/HER2+  N (%) | HR-/HER2-  N (%) |
| --- | --- | --- | --- | --- |
| Neoadjuvant chemotherapy regimen | | | | |
| CAF, CAF/Taxol | 2 (0.2) | 1(0.2) | 0 | 0 |
| CEF, CEF/Taxol, Cyclophosphamide | 27 (2.8) | 10(2.2) | 8(2.0) | 3(0.6) |
| AC, AC/Capecitabine | 213 (21.9) | 45(9.9) | 40(10.2) | 136(25.0) |
| AC/Taxol, AC/Taxol/Capecitabine, AC/Taxol/Carboplatin | 714(73.5) | 208(45.8) | 154(39.3) | 383(70.4) |
| Taxol, Taxol/Carboplatin, Taxol/Adriamycin | 10(1.0) | 165(36.3) | 168(42.9) | 11(2.0) |
| Doxorubicin/Taxol, Doxorubicin/Taxol/Carboplatin, Doxorubicin/Taxol/Epirubicin | 6(0.6) | 25(5.5) | 22(5.6) | 9(1.7) |
| TC | 0 | 0 | 0 | 2(0.4) |
| Unknown | 16 | 15 | 15 | 7 |
| Neoadjuvant anti-HER2 therapy | | | | |
| No exposure | 878(100) | 0 | 0 | 506(100) |
| Trastuzumab | 0 | 269(58.2) | 207(51.6) | 0 |
| Trastuzumab/pertuzumab | 0 | 188(40.7) | 190(47.4) | 0 |
| Pertuzumab/T-DM1 | 0 | 5(1.1) | 4(1.0) | 0 |
| Unknown |  |  |  |  |
| RCB | | | | |
| RCB0, pathologic CR | 65(6.6) | 161(34.3) | 227(55.8) | 165(29.9) |
| RCB1 | 66(6.7) | 92(19.6) | 47(11.5) | 55(10.0) |
| RCB2 | 544(55.1) | 173(36.9) | 101(24.8) | 245(44.5) |
| RCB3 | 313(31.7) | 43(9.2) | 32(7.9) | 86(15.6) |
| Total patients | 988 | 469 | 407 | 551 |
