# Supplementary figures and images for: Prognostic value of residual cancer burden after neoadjuvant chemotherapy in breast cancer: a comprehensive subtype-specific analysis
Source: Sci Rep. 2025 Apr 22;15:13977. doi: 10.1038/s41598-025-98176-9 (PMC12015579; doi:10.1038/s41598-025-98176-9)

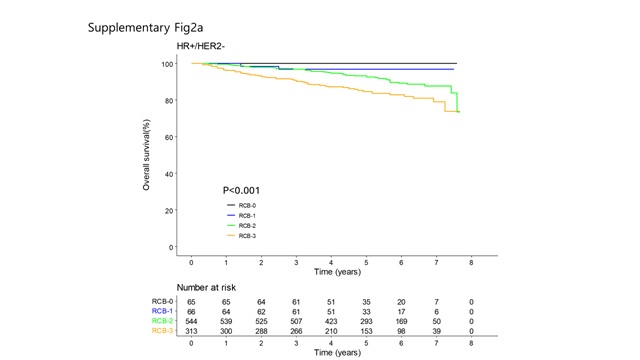

Supplement: Supplementary file 2 — Supplementary Material 2 [file 41598_2025_98176_MOESM2_ESM.jpg]

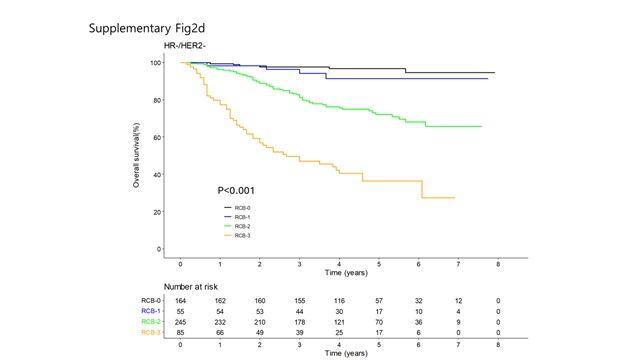

Supplement: Supplementary file 3 — Supplementary Material 3 [file 41598_2025_98176_MOESM3_ESM.jpg]

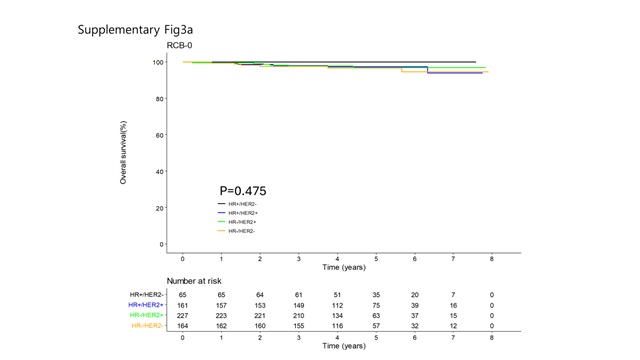

Supplement: Supplementary file 4 — Supplementary Material 4 [file 41598_2025_98176_MOESM4_ESM.jpg]

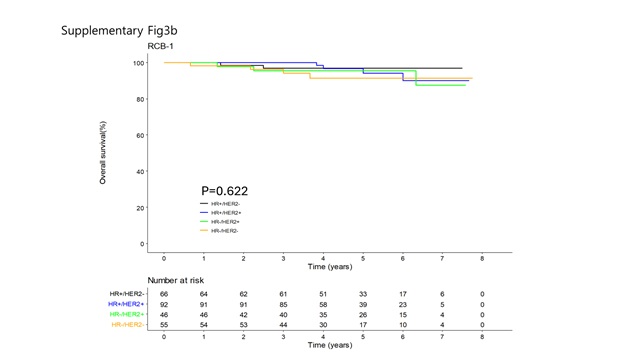

Supplement: Supplementary file 5 — Supplementary Material 5 [file 41598_2025_98176_MOESM5_ESM.jpg]

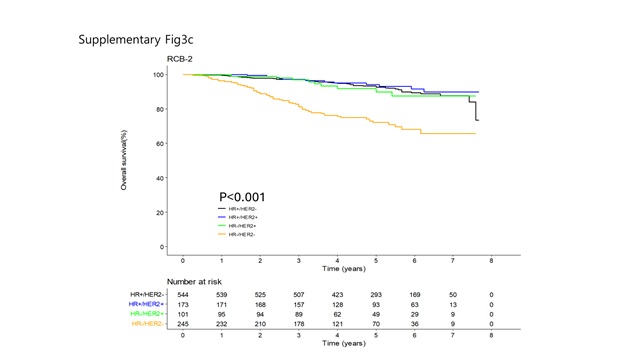

Supplement: Supplementary file 6 — Supplementary Material 6 [file 41598_2025_98176_MOESM6_ESM.jpg]

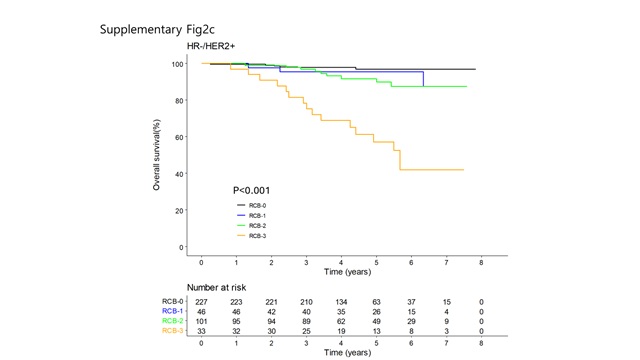

Supplement: Supplementary file 7 — Supplementary Material 7 [file 41598_2025_98176_MOESM7_ESM.jpg]

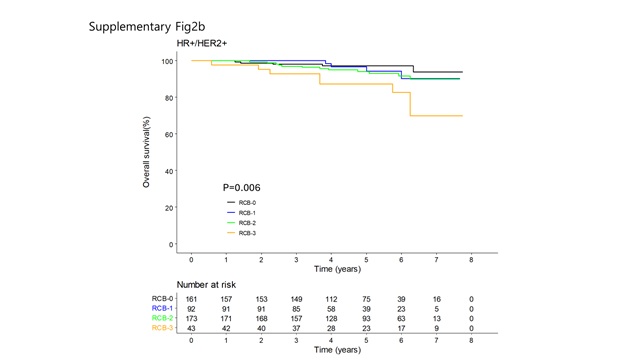

Supplement: Supplementary file 8 — Supplementary Material 8 [file 41598_2025_98176_MOESM8_ESM.jpg]

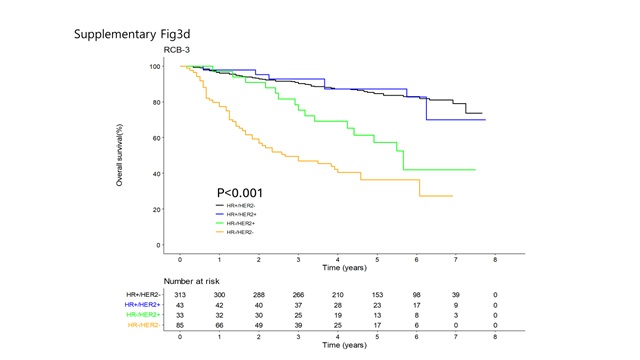

Supplement: Supplementary file 9 — Supplementary Material 9 [file 41598_2025_98176_MOESM9_ESM.jpg]
