# Supplementary material for: Prognostic value of residual cancer burden after neoadjuvant chemotherapy in breast cancer: a comprehensive subtype-specific analysis
Source: Sci Rep. 2025 Apr 22;15:13977. doi: 10.1038/s41598-025-98176-9 (PMC12015579; doi:10.1038/s41598-025-98176-9)
Supplement: Supplementary file 10 — Supplementary Material 10 [file 41598_2025_98176_MOESM10_ESM.docx]

**Supplementary Figure legends:**

**Supplementary Fig1. RCB formula**

CIS, carcinomain situ; CA, carcinoma

**Supplementary Fig2** Overall survival according to RCB classes and different subtypes. (a) HR+/HER2-; (b) HR+/HER2+; (c) HR-/HER2+; (d) HR-/HER2-

RCB, residual cancer burden; HR, hormone receptor; HER2, human epidermal growth factor receptor 2

**Supplementary Fig. 3** Overall survival across the different subtypes within each RCB classes. (a) RCB0; (b) RCB1; (c) RCB3; (d) RCB4

RCB, residual cancer burden
